# Hyperkalemia treatment modalities: A descriptive observational study focused on medication and healthcare resource utilization

**DOI:** 10.1371/journal.pone.0226844

**Published:** 2020-01-07

**Authors:** Nihar R. Desai, Christopher G. Rowan, Paula J. Alvarez, Jeanene Fogli, Robert D. Toto

**Affiliations:** 1 Internal Medicine, Center for Outcomes Research and Evaluation, Yale University, New Haven, Connecticut, United States of America; 2 Pharmacoepidemiology, COHRDATA, Santa Monica, California, United States of America; 3 Managed Care Health Outcomes, Relypsa, Inc., a Vifor Pharma Company, Redwood City, California, United States of America; 4 Medical Affairs, Relypsa, Inc., a Vifor Pharma Company, Redwood City, California, United States of America; 5 Internal Medicine, University of Texas Southwestern Medical Center, Dallas, Texas, United States of America; Universidade de Sao Paulo Faculdade de Medicina, BRAZIL

## Abstract

Renin-angiotensin-aldosterone system inhibitor (RAASi) therapy has been shown to improve outcomes among patients with congestive heart failure, diabetes, or renal dysfunction. These patients are also at risk for the development of hyperkalemia (HK), often leading to down-titration and/or discontinuation of RAASi therapy. Patiromer is the first sodium-free, non-absorbed potassium (K^+^) binder approved for the treatment of hyperkalemia (HK) in over 50 years. We described the association between use of K^+^ binders (Patiromer and sodium polystyrene sulfonate [SPS]) and renin-angiotensin-aldosterone system inhibitor (RAASi), on healthcare resource utilization (HRU). The study population consisted of Medicare Advantage patients with HK (K^+^ ≥ 5.0 mmol/L) in Optum’s Clinformatics^®^ Data Mart between 1/1/2016–12/31/2017. Patiromer and (SPS) initiators, and HK patients not exposed to a K^+^ binder (NoKb) were included. The index date was the date of the first K^+^ binder dispensing or HK diagnosis. Outcomes assessed at 6 months post-index were: (1) K^+^ binder utilization, (2) RAASi continuation, and (3) HRU (pre- vs post-index). HRU change was analyzed using McNemar’s statistical test. Study cohorts included 610 (patiromer), 5556 (SPS), and 21,282 (NoKb) patients. Overall baseline patient characteristics were: mean age 75 years; female 49%, low-income subsidy 29%, chronic kidney disease 48% (63% for patiromer cohort), and congestive heart failure 29%. At 6 months post-index, 28% (patiromer) and 2% (SPS) remained continuously exposed to the index K^+^ binder. RAASi continued for 78% (patiromer), 57% (SPS), and 57% (NoKb). The difference (pre- vs post-index) in hospitalized patients was: –9.4% (patiromer; *P*<0.05), –7.2% (SPS), and +16.8% (NoKb; *P*<0.001). Disparate K^+^ binder utilization patterns were observed. The majority of patiromer patients continued RAASi therapy while the percentage of SPS patients that continued RAASi therapy was lower, overlapping CIs were observed. Following continuous patiromer exposure, statistically significant reductions in hospital admissions and emergency department visits were observed, continuous SPS exposure observed no statistically significant reductions in either hospitalizations or ED visits, while NoKb patients with continuous exposure had statistically significant increases in both. Further research, with a larger sample size using comparative analytic methods, is warranted.

## Introduction

Hyperkalemia (HK) is a potentially life-threatening disorder due to alterations in cardiac conduction, which may result in arrhythmias and sudden death [1, 2]. The primary risk factor for HK is chronic kidney disease (CKD), due to decreased potassium (K^+^) excretion.[1, 3–8] Other risk factors for HK include diabetes, heart failure, and the use of renin-angiotensin-aldosterone system inhibitors (RAASi) [1, 2, 9, 10].

Despite the increased HK risk with RAASi use, RAASi utilization is critical to slowing CKD progression in certain patients—such as those with diabetes [2, 11–14]. RAASi therapy also reduces morbidity and mortality in patients with heart failure, [15, 16] improves clinical outcomes, and reduces healthcare costs [17, 18].

Pharmacologic options for the outpatient treatment of HK include sodium polystyrene sulfonate (SPS; Kayexalate^®^, Sanofi-Aventis U.S. LLC, Bridgewater, NJ, USA), a nonspecific sodium-cation exchange resin [2, 19]; patiromer; and sodium zirconium cyclosilicate (not approved at the time of this study). Limited evidence exists regarding the effectiveness of chronic SPS use—which may be limited by adverse events, including gastrointestinal symptoms in addition to other systemic toxicities, making it difficult for patients to tolerate long-term [2, 19].

Patiromer (VELTASSA^®^, Relypsa, Inc., a Vifor Pharma Group Company, Redwood City, CA, USA) was approved for the treatment of HK by the US Food and Drug Administration (FDA) in 2015 and by the European Medicines Agency in 2017 [2, 7, 8, 20]. Patiromer is a sodium-free non-absorbed polymer that exchanges calcium for K^+^, thus removing K^+^ from the body and lowering serum K^+^. Other small cations can also bind to patiromer but have a lower concentration in the colon than K^+^ [2, 20, 21]. In published randomized trials, patiromer’s efficacy was demonstrated in treating HK in patients with CKD, diabetes, hypertension, and heart failure, and in RAASi users. In one of the larger trials, patiromer resulted in a statistically significant reduction in serum K^+^ after 4 weeks that persisted with continued treatment through 52 weeks [2, 22–24].

Few studies have evaluated HK treatment modalities and RAASi utilization in real-world clinical practice. This descriptive study endeavored to address this evidence gap by: (*1*) describing K^+^ binder utilization, (*2*) describing RAASi continuation, and (*3*) describing pre- and post-index healthcare resource utilization (HRU).

## Methods

### Study design and data source

A retrospective, observational cohort study was conducted using “real-world” data from Optum’s Clinformatics Data Mart (Eden Prairie, MN, USA), which include administrative claims for individuals enrolled in commercial health insurance plans and Medicare Advantage insurance in the United States. The database contains individual-level, de-identified healthcare data on: patient enrollment, demographics, inpatient and outpatient medical claims, diagnostic and laboratory test claims, laboratory results for tests conducted at certain laboratories, and inpatient and outpatient medication claims. The medication data used in this study originated from outpatient pharmacy dispensing claims.

This retrospective administrative claims database analysis was based on historic deidentified patient data and did not involve patients directly; therefore, institutional review board/ethics committee approval was not necessary or applicable.

### Study cohorts and inclusion/exclusion criteria

During the 2-year study period (January 1, 2016, to December 31, 2017), patients with Medicare Advantage insurance were included in the patiromer and SPS cohorts who had at least one pharmacy dispensing claim for patiromer or SPS (identified using National Drug Codes [NDC]; S1 Table), respectively. Patients were included in the “no K^+^ binder” (NoKb) cohort who had at least one HK diagnosis code (identified using International Classification of Diseases, Ninth/Tenth Revisions, Clinical Modification [ICD-9/10-CM] codes: 276.7/E87.5). The index date was the date of the first patiromer/SPS dispensing claim during the study period or the date of the first HK diagnosis code (for the NoKb cohort). Patients who met the entry criteria for multiple cohorts were included in each cohort. The baseline period was 12 months before the index date (see study schema in S1 Fig). Patients were required to have continuous insurance enrollment during the last 6 months of the baseline period and a serum K^+^ test value ≥ 5.0 mmol/L within 3 months prior to the index date (using Logical Observation Identifier Names and Code [LOINC]: 2823–3).

### Follow-up and censoring

Follow-up began on the index date and continued until the first censoring event. Censoring events included: loss of insurance coverage (allowable ≤ 45-day coverage gap), death, and study period end date (ie, December 31, 2017). Intention-to-treat (ITT) analyses were based on these censoring criteria. Continuous exposure (CE) analyses additionally censored follow-up at the time of discontinuing the index K^+^ binder or switching among exposure groups. For the NoKb cohort, CE was censored if patients initiated either patiromer or SPS.

The rationale for using both ITT and CE exposure classifications was to assess the exposure-outcomes associations under “real-world” conditions where disparate K^+^ binder utilization exists; and to account for the heterogeneous clinical nature of HK, which may be acute, intermittent, or chronic.

Index K^+^ binder discontinuation was classified when the “days supplied” for the initial dispensing and all subsequent dispensings were exhausted with no subsequent dispensings within 31 days following the exhausted “days supplied.” Switching the index K^+^ binder was classified on the dispensing date for the opposing exposure status.

### Exposure classification

Exposure was classified as of the index date and continued until the first censoring event. As described above, exposure was classified using both ITT and CE approaches.

### Patiromer and SPS utilization outcome

The duration of the first continuous exposure episode was assessed using a Kaplan-Meier survival analysis for patients in the patiromer and SPS cohorts. CE began on the index date and continued until the first censoring event or discontinuing/switching exposure status. We also described the starting dosage and the percentage of patients with a dose increase/decrease.

### RAASi continuation outcome

RAASi continuation was assessed at 6 months post-index for patients who were continuously exposed to a RAASi during the last 6 months of the baseline period including the index date (using an allowable ≤30-day RAASi therapy gap). RAASi continuation was classified using pharmacy dispensing claims data (using NDC codes) for: angiotensin-converting-enzyme (ACE) inhibitors, angiotensin II receptor blockers (ARB), mineralocorticoid receptor antagonists (MRA), and direct renin inhibitors (DRI). Patients were included in these analyses who remained uncensored at 6 months post-index. The percentage of patients (with exact binomial 95% confidence interval [CI]) who continued RAASi therapy was assessed for both ITT and CE exposure classifications.

### HRU outcomes

HRU outcomes were assessed for patients in the patiromer, SPS, and NoKb cohorts at 6 months pre- and post-index. To evaluate equivalent outcome ascertainment periods, patients were included in the analysis set who remained uncensored at 6 months post-index. The HRU outcome metrics included: (*1*) the rate of pre- and post-index hospital admissions/emergency department (ED) visits, (*2*) the rate difference, and (*3*) the difference in the percentage of patients with at least one pre-index hospitalization/ED visit compared to the percentage of patients with at least one post-index hospitalization/ED visit.

Hospital admissions and ED visits associated with any medical diagnosis (ie, “all cause”) were classified on the admission/ED date using facility claims for inpatient admission or ED services. Admission dates which occurred during the length of stay of a prior admission were considered part of the same hospitalization. The percentage of patients with at least one admission/ED visit was also determined. HRU outcomes were assessed using both ITT and CE exposure classifications.

### Baseline patient characteristics

Comorbidities were classified using ICD-9/10-CM codes (S2 Table). Medications were classified using pharmacy dispensing claims data (using NDC codes). Serum K^+^ and estimated glomerular filtration rate (eGFR; Mayo Clinical Quadratic formula [serum creatinine LOINC Code: 2160–0]) were classified using laboratory results data. Comorbidities and medications were defined by the presence of at least one diagnosis code or medication dispensing, respectively, in the 12-month baseline period. Serum K^+^ and eGFR were classified using the last test value 3 months before the index date.

### Statistical analysis

Baseline characteristics were summarized as means with standard deviations (SD) for continuous variables, and the number of patients and percentages for categorical variables. Time to discontinuation of the first patiromer and SPS CE episode was determined using a Kaplan-Meier survival analysis. The rate of admissions/ED visits was defined as the quotient of the total number of “events” (ie, total number of admissions or ED visits) and person-time (standardized to person-years). The rate difference and 95% CI were calculated as the post-index rate minus the pre-index rate. McNemar’s chi-squared test for binomial paired data (ie, pre- vs post-index events for patients in the same study cohort) was used to analyze the proportion of patients with a hospital admission or ED visit pre- vs post-index. All statistical analyses were conducted using STATA Version 15 (StataCorp, LLC, College Station, TX, USA).

For a variety of reasons related to medication adherence, pharmacy dispensing data potentially have incomplete ascertainment of medication use and discontinuation. This missing data problem was addressed using both ITT and CE classifications. The ITT exposure classification addresses the potential missing data issue by attributing all follow-up time from the index date to the first censoring event (excluding censoring events related to medication discontinuation or switching). All patients were required to have a baseline serum K^+^; therefore, both treatment groups will have complete ascertainment of baseline serum K^+^. Binary baseline variables and binary outcomes (e.g., hospitalization, ED visits, and RAASi use) were coded as present (1) or absent (0). No evidence of hospitalization, ED visit, or a RAASi dispensing in the baseline period or during follow-up were classified as “no” or absent (0).

## Results

### Characteristics of the study cohorts

A total of 25,197 patients met the study inclusion criteria, with 610 in the patiromer cohort, 5556 in the SPS cohort, and 21,282 in the NoKb cohort. S3 Table shows details of patients included/excluded by each criterion. The Venn diagram in S2 Fig shows the overlap of patients included in multiple study cohorts.

Overall, 65% of patiromer, 73% of SPS, and 87% of NoKb patients were excluded from the initial cohort entry criterion (ie, patiromer/SPS dispensing or HK diagnosis in the study period). The criterion resulting in the greatest exclusion of patients was not having a baseline serum K^+^ available (patients excluded: patiromer = 45%, SPS = 59%, NoKb = 68%). This finding is consistent with the current availability of laboratory data among patients in Optum’s Clinformatics Data Mart (estimated to be ~30% of all patients). Among patients with an available baseline serum K^+^, 9% of patients in the patiromer and SPS cohorts and 33% of patients in the NoKb cohort did meet the requirement for a baseline serum K^+^ ≥5.0 mmol/L.

The follow-up attrition table shows the number of patients included in the 6-month CE and ITT analyses and the censoring reason (S4 Table).

### Baseline patient characteristics

Baseline demographic and clinical characteristics are presented in Table 1. The median age and sex distributions were similar. Low-income subsidy was present for fewer patients in the NoKb cohort. Mean baseline serum K^+^ was similar (5.5–5.8 mmol/L) among all cohorts, however baseline serum K^+^ > 6.0 mmol/L was greater among the SPS cohort. Patiromer initiators were more likely to have baseline CKD and lower eGFR; however, less likely to have had baseline congestive heart failure. Forty-five percent (45%) of patiromer initiators had a baseline SPS dispensing, compared to 6% in the SPS and NoKb cohorts. Baseline RAASi utilization was similar (64–72%), but utilization of loop/thiazide diuretics and insulin was higher among patients in the patiromer cohort.

**Table 1:** Baseline patient characteristics.

	Patiromer	SPS	No K^+^ Binder
	n = 610	n = 5556	n = 21,282
**Demographics**			
Mean age, years (SD)	74 (9)	75 (9)	74 (9)
Female, n (%)	253 (41)	2654 (48)	10,485 (49)
Low income subsidy, n (%)	227 (37)	2096 (38)	5651 (27)
**Comorbidities (12 months before index date), n (%)**			
CKD	386 (63)	2804 (50)	9999 (47)
ESRD	27 (4)	165 (3)	555 (3)
Congestive heart failure	110 (18)	1155 (21)	5105 (24)
Cancer	48 (8)	609 (11)	2894 (14)
Diabetes mellitus	269 (44)	2351 (42)	10,415 (49)
Cerebrovascular disease	63 (10)	590 (11)	2570 (12)
Myocardial infarction	57 (9)	649 (12)	2941 (14)
Cardiac dysrhythmias	110 (18)	1142 (21)	5711 (27)
Coronary artery disease	160 (26)	1544 (28)	7090 (33)
**Baseline medications (12 months before index date), n (%)**			
SPS	275 (45)	351 (6)	1186 (6)
RAASi therapy	421 (69)	3980 (72)	13,535 (64)
ACE inhibitor	230 (38)	2587 (47)	8898 (42)
ARB	205 (34)	1497 (27)	4712 (22)
MRA	45 (7)	593 (11)	2183 (10)
Loop diuretic	324 (53)	2261 (41)	6430 (30)
Thiazide	134 (22)	1129 (20)	3899 (18)
Insulin	232 (38)	1657 (30)	4223 (20)
**Baseline serum K**^**+**^ **and eGFR (3 months before index date)**			
Mean serum K^+^, mmol/L (SD)	5.6 (0.4)	5.8 (0.5)	5.5 (0.5)
K^+^, n (%)			
5.0 to < 5.5 mmol/L	256 (42)	1157 (21)	10,873 (51)
5.5 to < 6.0 mmol/L	269 (44)	2534 (46)	7391 (35)
6.0 to < 6.5 mmol/L	67 (11)	1436 (26)	2064 (10)
≥ 6.5 mmol/L	18 (3)	429 (8)	954 (4)
Mean eGFR mL/min/1.73m^2^ (SD)	33 (20)	45 (26)	56 (28)
eGFR, n (%)			
≥90 mL/min/1.73m^2^	7 (1)	331 (6)	2394 (12)
60 to 89 mL/min/1.73m^2^	62 (10)	1125 (21)	6708 (34)
30 to 59 mL/min/1.73m^2^	196 (32)	1885 (35)	6317 (32)
15 to 29 mL/min/1.73m^2^	231 (38)	1412 (27)	3079 (15)
<15 mL/min/1.73m^2^	111 (18)	567 (11)	1391 (7)

ACE, angiotensin-converting enzyme; ARB, angiotensin II receptor blocker; CKD, chronic kidney disease; eGFR, estimated glomerular filtration rate; ESRD, end-stage renal disease; K^+^ potassium; MRA, mineralocorticoid receptor antagonist; RAASi, renin-angiotensin-aldosterone inhibitor; SD, standard deviation; SPS, sodium polystyrene sulfonate.

### Patiromer and SPS utilization results

**Fig 1:**
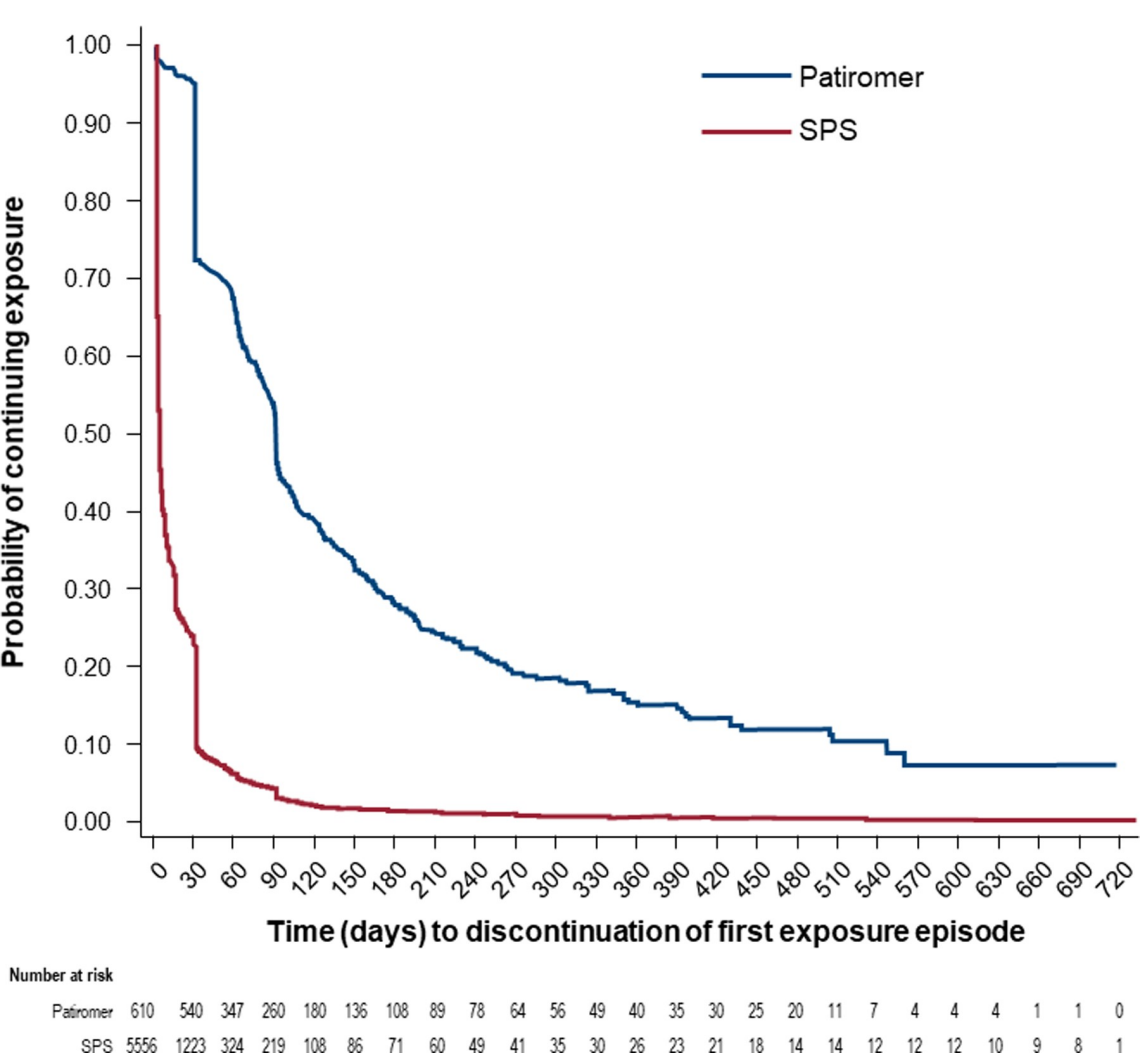
Duration of continuous K^+^ binder use (patiromer and SPS) to the first censoring event. Censoring events: index K^+^ binder discontinuation, opposing K^+^ binder dispensing, insurance disenrollment, end of study period (12/31/2017) Death. K^+^, potassium; SPS, sodium polystyrene sulfonate.

The initial patiromer and SPS dosage was 8.4 g and 15 g for 95% and 71%, respectively. Fig 1 shows the Kaplan-Meier curve for continuous exposure to patiromer and SPS. For the patiromer cohort, the percentage of patients continuously exposed at 30, 90, and 180 days post-index was 72%, 50%, and 28%, respectively. For the SPS cohort, the percentage of patients continuously exposed at 30, 90, and 180 days was 20%, 5%, and 2%, respectively. Within 6 months post-index, the median (p25, p75) number of patiromer and SPS dispensings was 3 (2, 5) and 1 (1, 1), respectively; dose increases and decreases were uncommon (<10%) in both groups.

### RAASi continuation results

Continuous baseline RAASi utilization was identified for 214 (35%), 2371 (43%), and 8531 (40%) of patients in the patiromer, SPS, and NoKb cohorts, respectively. At 6 months post-index, 36 (patiromer), 35 (SPS), and 5127 (NoKb) patients were included in the CE analyses; 102 (patiromer), 1627 (SPS), and 5543 (NoKb) were included in the ITT analyses.

**Fig 2:**
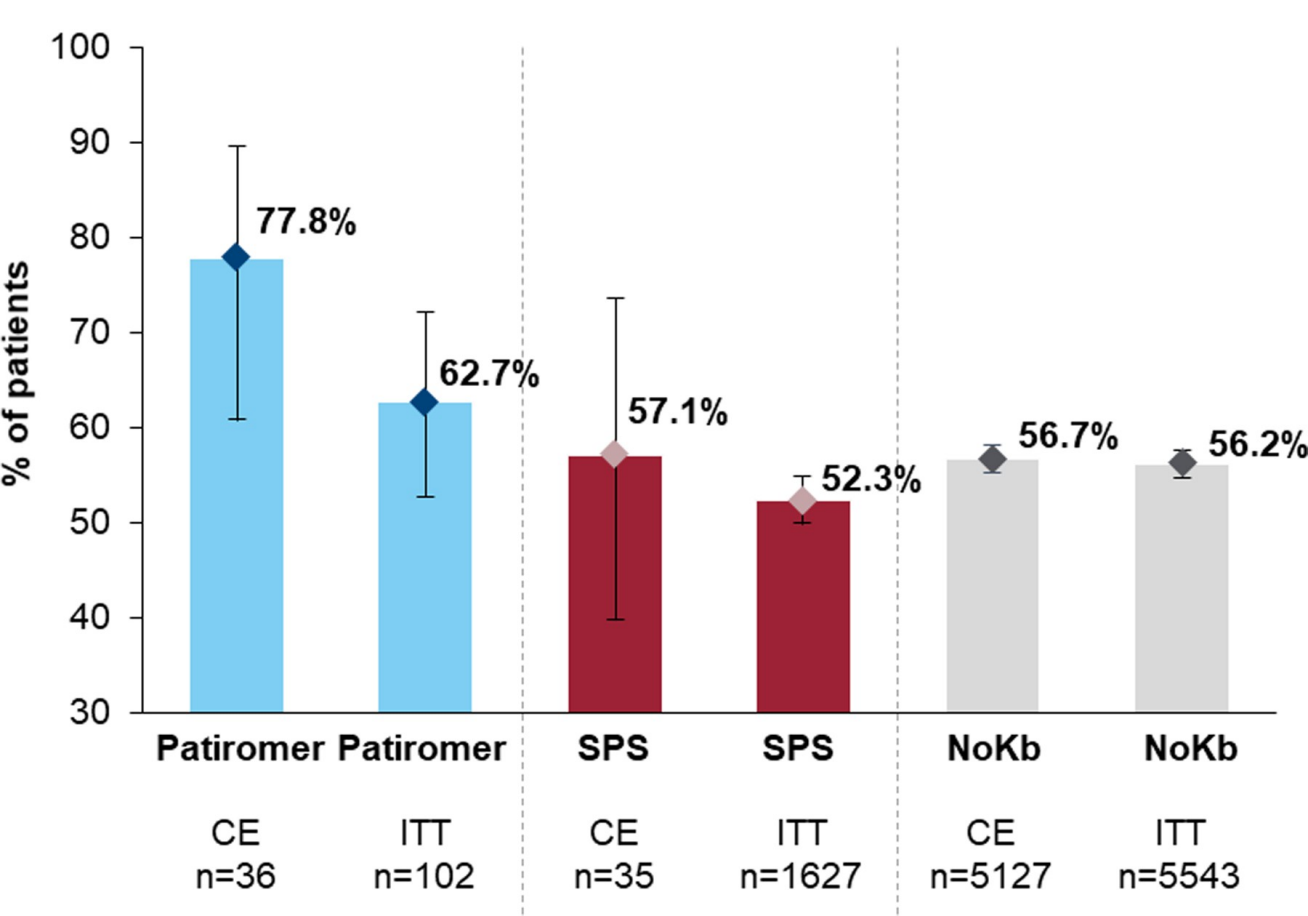
Percentage (95% CI) of patients continuing RAASi* therapy: By treatment group and exposure classification (CE, ITT). *RAASi included: ACE inhibitors, ARBs, MRAs, and DRIs. CE, continuous exposure; CI, confidence interval; ITT, intention-to-treat; NoKb, no potassium binder; RAASi, renin-angiotensin-aldosterone system inhibitor; SPS, sodium polystyrene sulfonate.

Fig 2 shows the percentage (and 95% CI) of patients who remained continuously exposed to RAASi therapy from the index date through 6 months post-index. Overlapping CIs were observed for patiromer and SPS. Despite overlapping CIs, patients continuously exposed to a K^+^ binder included a numerically greater percentage of patients who also continued RAASi therapy (patiromer 78% [95% CI: 61–90]; SPS 57% [95% CI: 39–74]) compared to patients in the ITT exposure analyses (patiromer 63% [95% CI: 53–72]; SPS 52% [95% CI: 50–55]). The percentage of patients who continued RAASi therapy for the NoKb cohort was approximately 56% for both CE and ITT exposure classifications.

### HRU results

Table 2 shows the pre- and post-index hospital admission and ED visit rates. At 6 months post-index, 106 (patiromer), 69 (SPS), and 12,596 (NoKb) patients were included in the CE analyses; 339 (patiromer), 3785 (SPS), and 13,598 (NoKb) patients were included in the ITT analyses.

**Table 2:** ED visit rate and hospitalization rate: 6 months pre-index and 6 months post-index: By treatment group and exposure classification.

	n^a^ pre- and post-index	Person-years pre- and post-index	Number of patients with ≥ 1 event		Total number of events		Event rate^b^ (per person-years)		Rate difference (post-index– pre-index) (95% CI)
			Pre-index	Post-index	Pre-index	Post-index	Pre-index	Post-index	
***ED visits***									
CE^c^									
Patiromer	106	53	30	17	53	21	1.00	0.40	–0.60 (–0.92, –0.29)
SPS	69	35	13	13	25	22	0.72	0.64	–0.08 (–0.47, 0.30)
NoKb	12,596	6298	3766	5647	6725	11,407	1.07	1.81	0.74 (0.70, 0.78)
ITT^d^									
Patiromer	339	170	76	67	130	111	0.77	0.65	–0.12 (–0.29, 0.07)
SPS	3785	1893	1006	990	1816	1868	0.96	0.99	0.03 (–0.03, 0.09)
NoKb	13,598	6799	4086	6127	7262	12,384	1.07	1.82	0.75 (0.71, 0.79)
***Hospital admissions***									
CE									
Patiromer	106	53	17	7	22	9	0.42	0.17	–0.25 (–0.45, -0.04)
SPS	69	35	11	6	16	8	0.46	0.23	–0.23 (–0.50, 0.05)
NoKb	12,596	6298	2171	4288	3375	7598	0.54	1.21	0.67 (0.64, 0.70)
ITT									
Patiromer	339	170	46	46	61	63	0.36	0.37	0.01 (–0.12, 0.14)
SPS	3785	1893	665	616	1019	1024	0.54	0.54	0.00 (–0.04, 0.05)
NoKb	13,598	6799	2363	4620	3655	8152	0.54	1.2	0.66 (0.63, 0.69)

CE, continuous exposure; CI, confidence interval; ED, emergency department; ITT, intention-to-treat; K^+^, potassium; NoKb, no K^+^ binder; SPS, sodium polystyrene sulfonate.

^a^ n is the number of patients who remained uncensored at 6 months post-index and were included in the 6 month pre- and post-index analysis.

^b^ The event rate is the number of events (ED visits or hospital admissions) in the 6 month pre-index and post-index periods, standardized to person-years.

^c^ Patients in the patiromer and SPS cohorts were continuously exposed to either K^+^ binder from the index date through 6 months post-index. Patients in the NoKb cohort did not initiate either patiromer or SPS from the index date through 6 months post-index.

^d^ Patients in all three cohorts began in their assigned cohort as of the index date; however, their exposure status may have changed during the 6-month post-index period.

For the CE analyses, the ED visits rate ranged from 0.7–1.1 (per person-years) pre-index and 0.4–1.8 post-index (Table 2). The ED visit rate decreased following continuous K+ binder exposure (patiromer: –0.60 [95% CI: –0.92, –0.29]; SPS: –0.08 [95% CI: –0.47, 0.30]) and increased for patients who did not initiate a K+ binder exposure (NoKb: +0.74 [95% CI: 0.70, 0.78]). A similar pattern was observed for hospital admissions, although the absolute hospital admission rates were roughly half that of ED visit rates. The pre- vs. post-index change in hospital admission rates (ie, the rate difference) was comparable between the patiromer (–0.25 [95% CI: –0.45, –0.04]) and SPS groups (–0.23 [95% CI: –0.50, 0.05]). However, in the NoKb group, hospital admission rates increased. For the ITT analyses, the ED and hospital admission rate differences were attenuated for patients in the patiromer and SPS cohorts.

**Fig 3:**
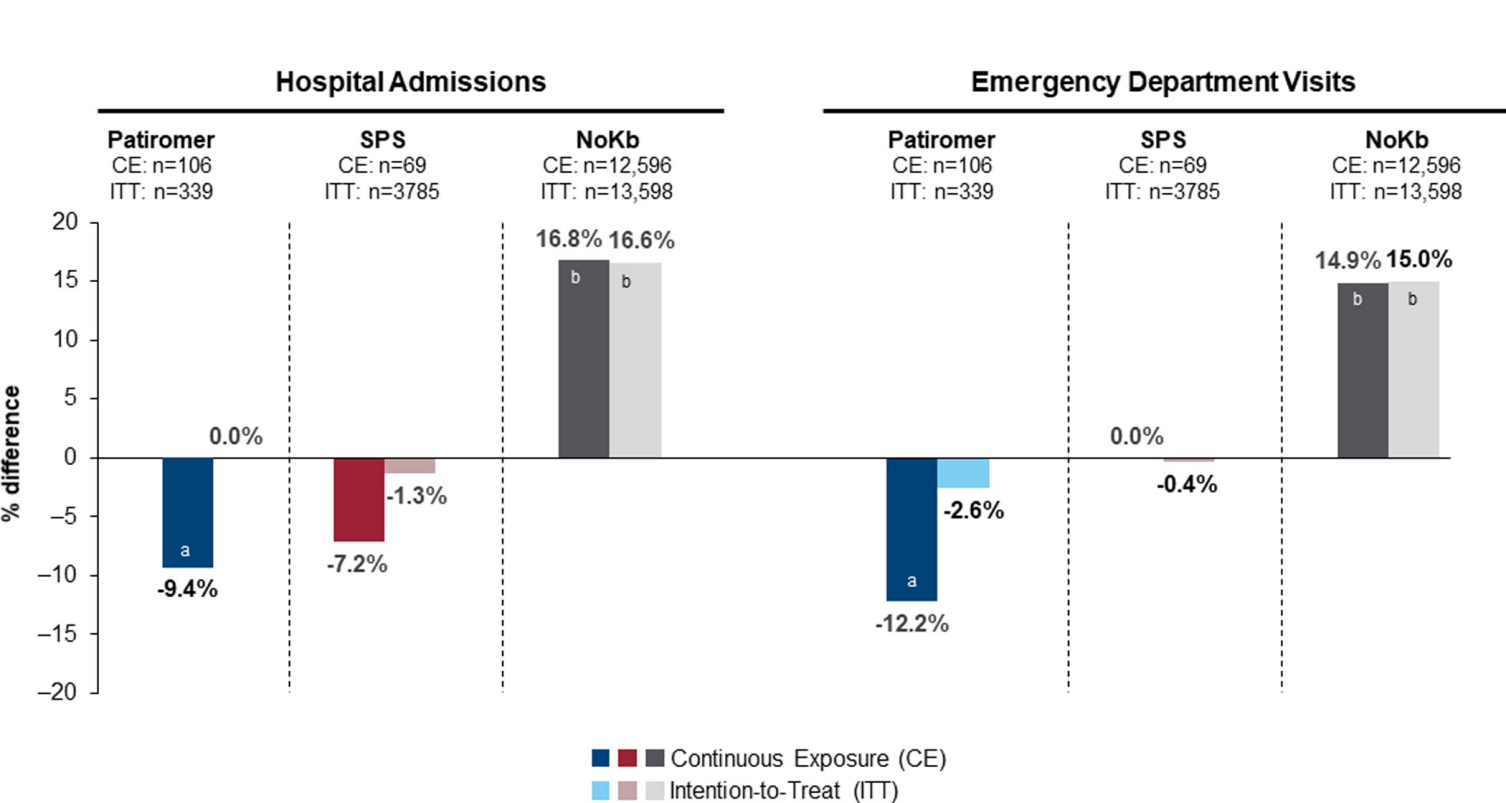
Change in percentage of patients (pre- vs post-index) with at least one hospital admission or ED visit: By treatment group and exposure classification (CE, ITT). Analyzed using McNemar’s test for paired nominal data (% post-index–% pre-index): ^a^*P*<0.05, ^b^*P*<0.001. CE, continuous exposure; ITT, intention-to-treat; SPS, sodium polystyrene sulfonate.

Fig 3 shows results for the change in the percentage of patients with an event (admission or ED visit) within 6 months post-index compared to 6 months pre-index. Following continuous patiromer exposure, the paired analyses showed statistically significant reductions in patients hospitalized (–9.4%; *P*<0.05) and with an ED visit (–12.2%; *P*<0.05). With SPS continuous exposure, the paired analyses did not show statistically significant reductions in patients hospitalized (-7.4%; *P*>0.05) and with ED visit no change was observed. For HK patients not exposed to a K^+^ binder (the NoKb cohort), significant increases in the percentage of patients with a hospital admission (~17%; *P*<0.001) and ED visit (~15%; *P*<0.001) were observed.

## Discussion

Hyperkalemia is one of the major reasons for guideline-recommended ACE inhibitors and ARBs are discontinued or fail to reach guideline-recommended dosing for CKD and/or diabetic nephropathy patients ([18, 25]). Until a few years ago, there was limited support for chronic use of potassium binders to treat HK with concurrent RAASi use, but with the newer binders, such as patiromer, the clinical evidence in maintaining normokalemia chronically is now available [24]. Our study is the first to evaluate HK treatment modalities and RAASi utilization in the real world setting.

This descriptive study, in the 2 years following the FDA approval of patiromer, shows that early patiromer initiators had more severe CKD and recent SPS use compared to patients in the SPS and NoKb cohorts, despite similar baseline serum K^+^ values. It is important to acknowledge that while the baseline serum K^+^ values were similar, the SPS cohort had more patients with baseline serum K^+^ > 6.0 mmol/L than the other cohorts. Disparate utilization patterns were observed for patiromer- and SPS-exposed patients. The Kaplan-Meier analysis depicts longer continuous patiromer utilization—perhaps suggesting the intention for chronic use. This contrasts with the apparent episodic or acute use of SPS, where only 25% of patients remained continuously exposed at 1 month post-index.

Among patients continuously exposed to patiromer and SPS for 6 months, 78% and 57%, respectively, also remained continuously exposed to RAASi therapy. The rate of hospital admissions and ED visits decreased following continuous exposure to a K^+^ binder (the patiromer and SPS cohorts) and increased for patients not exposed to a K^+^ binder (the NoKb cohort). No statistically significant reductions in hospital admissions or ED visits were seen for patients with continuous SPS exposure, only a numerical reduction in hospitalizations. Statistically significant reductions in hospital admissions and ED visits were observed for patients following continuous patiromer exposure in this descriptive analysis.

Comparing the results of the present study to other published studies is challenging since, as of the development of this manuscript, no other studies have been published evaluating K^+^ binder utilization, RAASi continuation, or HRU in a “real-world” cohort of Medicare Advantage patients with HK. Nevertheless, consistent with clinical trial findings,^21^ the present study demonstrates that patients continuously exposed to patiromer for 6 months also had high RAASi continuation rates. In an observational study of patients with HK, [26] the 1-year hospital admission and ED visit rates were 0.44 and 0.86, respectively. These were consistent with the baseline (ie, 6 months pre-index) rates in the present study, though substantially higher than the observed hospital admission and ED visit rates following 6 months of CE to patiromer (0.17 admission rate; 0.40 ED rate) and SPS (0.23 admission rate; 0.64 ED rate).

Our study was a descriptive analysis, so no matching of baseline characteristics were done. There are several important limitations for the study. First, while patient selection was broadly inclusive of Medicare Advantage patients with HK in a real-world setting, a large number of patients (50–80%) were excluded by requiring a baseline serum K^+^ ≥5.0 mmol/L. Although this may impact generalizability to some degree, the inclusion of patients with known HK is critical to safeguarding internal validity. Despite the fact that pharmacy dispensing data have been shown to reliably predict medication exposure in published validation studies, [27–32] exposure misclassification related to non-adherence or medication discontinuation may have occurred for a variety of reasons. To evaluate this potential limitation, we used both ITT and CE classifications. The results show magnified effect estimates in the CE analyses (for patiromer and SPS) compared to the attenuated findings in the ITT analyses. Thus, the observed differential treatment effects in the CE analyses compared to the ITT analyses suggest that the impact of exposure misclassification was minimal. For the RAASi continuation analyses, limited precision is evident in the wide CIs (Fig 2). However, limited precision is not unexpected as these analyses required continuous baseline RAASi exposure, which reduced the study population by over 50%. Further research in a larger study population is merited. The single-arm, within-patient (ie, pre-vs-post) HRU analyses, while valid in their current form, do not provide causal conclusions regarding the comparative effectiveness of patiromer compared to other study cohorts.

## Conclusion

This descriptive observational study among Medicare Advantage patients with HK showed disparate utilization patterns for patiromer and SPS, with longer duration associated with patiromer use. Continuous patiromer exposure was associated with a relatively high rate of RAASi therapy continuation and decreased rates of hospitalizations and ED visits, while SPS continuous exposure was not. Further research, with a larger sample size and comparative analytic methods, is warranted to fully elucidate these findings and generate causal estimates.

## Supporting information

S1 Fig Study schema. RAASI, renin-angiotensin-aldosterone system inhibitor; SPS, sodium polystyrene sulfonate.
(TIF)

S2 Fig Venn diagram of patients included in each study cohort. NoKb, no potassium binder; SPS, sodium polystyrene sulfonate.
(TIF)

S1 Table NDC for patiromer and SPS. NDC, National Drug Codes; SORB, sorbitex; SPS, sodium polystyrene sulfonate.
(DOCX)

S2 Table ICD-9/10-CM codes to classify baseline characteristics. CM, Clinical Modification; ICD-9, International Classification of Diseases, Ninth Revision; ICD-10, ICD, Tenth Revision.
(DOCX)

S3 Table Patients included/excluded from cohort entry. NoKb, no potassium binder; SPS, sodium polystyrene sulfonate.
(DOCX)

S4 Table Attrition table at 6 months post-index. b/f fu6, before the 6-month follow-up; K^+^, potassium; NoKb, no K^+^ binder; SPS, sodium polystyrene sulfonate.
(DOCX)
