# Supplementary figures and images for: Hyperkalemia treatment modalities: A descriptive observational study focused on medication and healthcare resource utilization
Source: PLoS One. 2020 Jan 7;15(1):e0226844. doi: 10.1371/journal.pone.0226844 (PMC6946143; doi:10.1371/journal.pone.0226844)

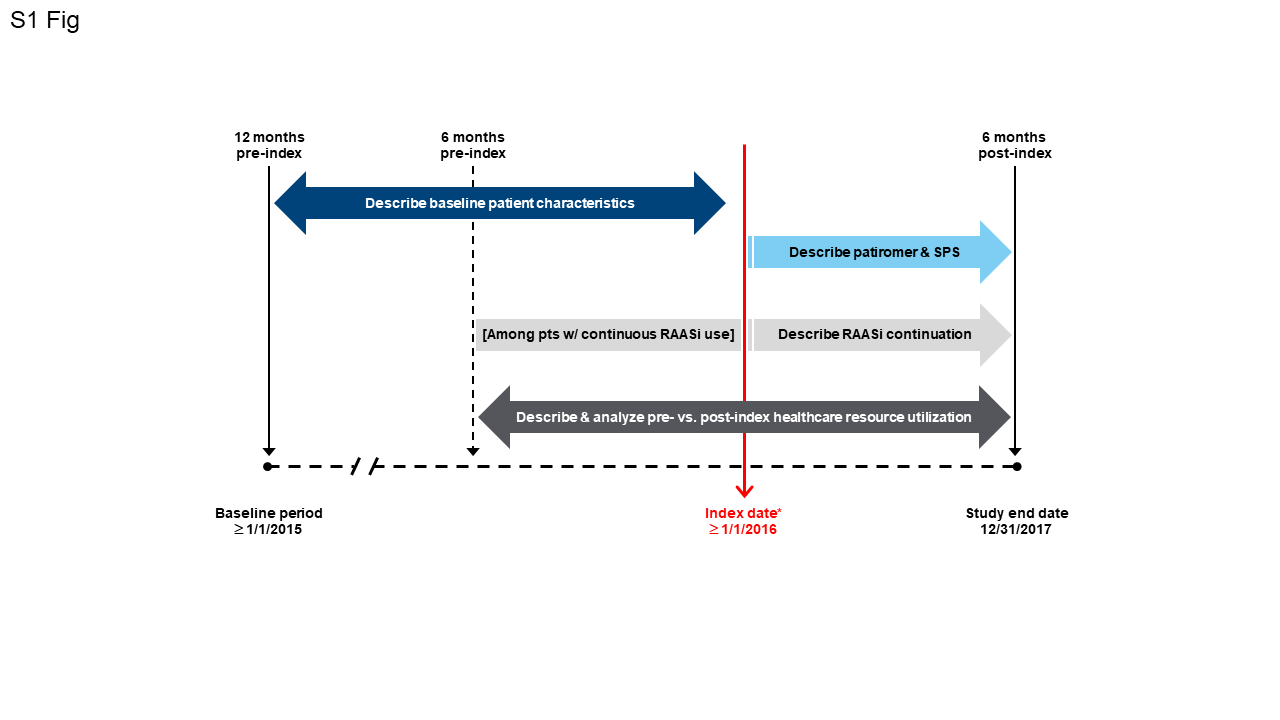

Supplement: S1 Fig — RAASI, renin-angiotensin-aldosterone system inhibitor; SPS, sodium polystyrene sulfonate. (TIF) [file pone.0226844.s001.tif]

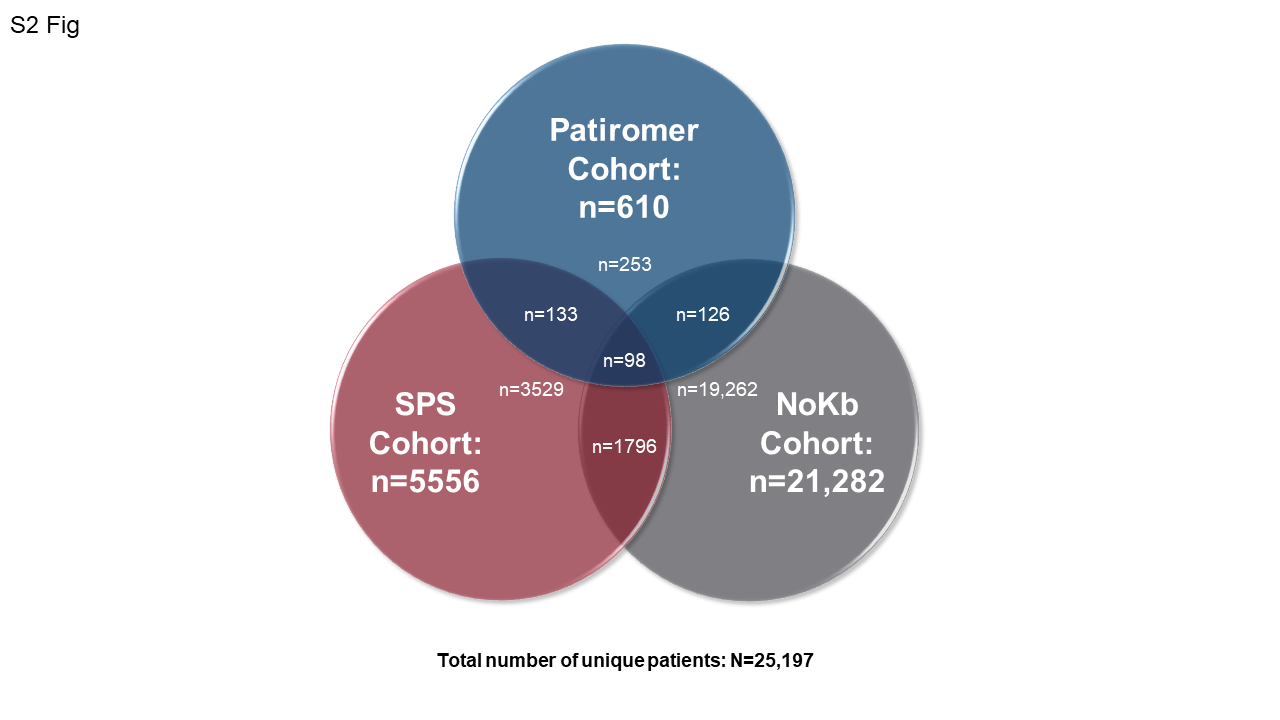

Supplement: S2 Fig — NoKb, no potassium binder; SPS, sodium polystyrene sulfonate. (TIF) [file pone.0226844.s002.tif]
