# Supplementary material for: Hyperkalemia treatment modalities: A descriptive observational study focused on medication and healthcare resource utilization
Source: PLoS One. 2020 Jan 7;15(1):e0226844. doi: 10.1371/journal.pone.0226844 (PMC6946143; doi:10.1371/journal.pone.0226844)
Supplement: S1 Table — NDC, National Drug Codes; SORB, sorbitex; SPS, sodium polystyrene sulfonate. (DOCX) [file pone.0226844.s003.docx]

# S1 Table. NDC for patiromer and SPS.

| **Brand Name** | **Generic Name** | **NDC Code** |
| --- | --- | --- |
| Kalexate | Sodium polystyrene sulfonate | 10702003615 |
| Kalexate | Sodium polystyrene sulfonate | 10702003645 |
| Kalexate | Sodium polystyrene sulfonate | 17856003615 |
| Kayexalate | Sodium polystyrene sulfonate | 58016761301 |
| Kayexalate | Sodium polystyrene sulfonate | 24107501 |
| Kayexalate | Sodium polystyrene sulfonate | 24987007501 |
| Kionex | Sodium polystyrene sulfonate | 574200416 |
| Kionex | Sodium polystyrene sulfonate | 574200216 |
| Kionex | Sodium polystyrene sulfonate | 574200202 |
| Kionex | Sodium polystyrene sulfonate/SORB | 574200216 |
| Kionex | Sodium polystyrene sulfonate/SORB | 574200202 |
| Marlexate™ | Sodium polystyrene sulfonate | 10135014617 |
| Sodium polystyrene sulfonate | Sodium polystyrene sulfonate | 54881755 |
| Sodium polystyrene sulfonate | Sodium polystyrene sulfonate | 69315020545 |
| Sodium polystyrene sulfonate | Sodium polystyrene sulfonate | 10702003615 |
| Sodium polystyrene sulfonate | Sodium polystyrene sulfonate | 686001216 |
| Sodium polystyrene sulfonate | Sodium polystyrene sulfonate | 54037963 |
| Sodium polystyrene sulfonate | Sodium polystyrene sulfonate | 574200302 |
| Sodium polystyrene sulfonate | Sodium polystyrene sulfonate | 54881501 |
| Sodium polystyrene sulfonate | Sodium polystyrene sulfonate | 54016550 |
| Sodium polystyrene sulfonate | Sodium polystyrene sulfonate | 38779025808 |
| Sodium polystyrene sulfonate | Sodium polystyrene sulfonate | 38779025850 |
| Sodium polystyrene sulfonate | Sodium polystyrene sulfonate | 54016555 |
| Sodium polystyrene sulfonate | Sodium polystyrene sulfonate | 10702003645 |
| Sodium polystyrene sulfonate | Sodium polystyrene sulfonate | 54016551 |
| Sodium polystyrene sulfonate | Sodium polystyrene sulfonate | 46287001216 |
| Sodium polystyrene sulfonate | Sodium polystyrene sulfonate | 54380563 |
| Sodium polystyrene sulfonate | Sodium polystyrene sulfonate | 54016563 |
| Sodium polystyrene sulfonate | Sodium polystyrene sulfonate | 574200316 |
| Sodium polystyrene sulfonate | Sodium polystyrene sulfonate | 42808050016 |
| Sodium polystyrene sulfonate | Sodium polystyrene sulfonate | 54037955 |
| Sodium polystyrene sulfonate | Sodium polystyrene sulfonate | 49452694503 |
| Sodium polystyrene sulfonate | Sodium polystyrene sulfonate | 10135014614 |
| Sodium polystyrene sulfonate | Sodium polystyrene sulfonate | 38779025854 |
| Sodium polystyrene sulfonate | Sodium polystyrene sulfonate | 54868443900 |
| Sodium polystyrene sulfonate | Sodium polystyrene sulfonate | 49452694501 |
| Sodium polystyrene sulfonate | Sodium polystyrene sulfonate | 11534016644 |
| Sodium polystyrene sulfonate | Sodium polystyrene sulfonate | 54037950 |
| Sodium polystyrene sulfonate | Sodium polystyrene sulfonate | 54037951 |
| Sodium polystyrene sulfonate | Sodium polystyrene sulfonate | 49452694502 |
| Sodium polystyrene sulfonate | Sodium polystyrene sulfonate | 54881611 |
| Sodium polystyrene sulfonate | Sodium polystyrene sulfonate | 69315020515 |
| Sodium polystyrene sulfonate | Sodium polystyrene sulfonate | 904604127 |
| Sodium polystyrene sulfonate | Sodium polystyrene sulfonate | 42806001396 |
| Sodium polystyrene sulfonate | Sodium polystyrene sulfonate | 832850616 |
| Sodium polystyrene sulfonate | Sodium polystyrene sulfonate | 10135014617 |
| Sodium polystyrene sulfonate | Sodium polystyrene sulfonate | 11534016615 |
| SPS | Sodium polystyrene sulfonate | 46287000601 |
| SPS | Sodium polystyrene sulfon/SORB | 46287000604 |
| SPS | Sodium polystyrene sulfonate | 46287000660 |
| SPS | Sodium polystyrene sulfonate | 46287000604 |
| SPS | Sodium polystyrene sulfon/SORB | 46287000660 |
| SPS | Sodium polystyrene sulfon/SORB | 46287000601 |
| VELTASSA^®^ | Patiromer calcium sorbitex | 53436008401 |
| VELTASSA^®^ | Patiromer calcium sorbitex | 53436025201 |
| VELTASSA^®^ | Patiromer calcium sorbitex | 53436016830 |
| VELTASSA^®^ | Patiromer calcium sorbitex | 53436025230 |
| VELTASSA^®^ | Patiromer calcium sorbitex | 53436008404 |
| VELTASSA^®^ | Patiromer calcium sorbitex | 53436008430 |
| VELTASSA^®^ | Patiromer calcium sorbitex | 53436016801 |

NDC, National Drug Codes; SORB, sorbitex; SPS, sodium polystyrene sulfonate.
