# Supplementary material for: Hyperkalemia treatment modalities: A descriptive observational study focused on medication and healthcare resource utilization
Source: PLoS One. 2020 Jan 7;15(1):e0226844. doi: 10.1371/journal.pone.0226844 (PMC6946143; doi:10.1371/journal.pone.0226844)
Supplement: S2 Table — CM, Clinical Modification; ICD-9, International Classification of Diseases, Ninth Revision; ICD-10, ICD, Tenth Revision. (DOCX) [file pone.0226844.s004.docx]

# S2 Table. ICD-9/10-CM codes to classify baseline characteristics.

| **Term Name** | **ICD-9/10-CM Code** | **Start** | **Stop** |
| --- | --- | --- | --- |
| Chronic kidney disease | ICD-9 Diagnosis | 585.2 | 585.5 |
| Chronic kidney disease | ICD-10 Diagnosis | N18.2 | N18.5 |
| Chronic kidney disease | ICD-9 Diagnosis | 585.9 | 585.9 |
| Chronic kidney disease | ICD-10 Diagnosis | N18.9 | N18.9 |
| End-stage renal disease | ICD-9 Diagnosis | 585.6 | 585.6 |
| End-stage renal disease | ICD-10 Diagnosis | N18.6 | N18.6 |
| Congestive heart failure | ICD-9 Diagnosis | 402.01 | 402.01 |
| Congestive heart failure | ICD-9 Diagnosis | 402.11 | 402.11 |
| Congestive heart failure | ICD-9 Diagnosis | 402.91 | 402.91 |
| Congestive heart failure | ICD-9 Diagnosis | 425 | 425.9 |
| Congestive heart failure | ICD-9 Diagnosis | 428 | 428.9 |
| Congestive heart failure | ICD-9 Diagnosis | 429.3 | 429.3 |
| Congestive heart failure | ICD-10 Diagnosis | I09.9 | I09.ZZZZ |
| Congestive heart failure | ICD-10 Diagnosis | I11.0 | I11.0ZZZ |
| Congestive heart failure | ICD-10 Diagnosis | I13.0 | I13.0ZZZ |
| Congestive heart failure | ICD-10 Diagnosis | I13.2 | I13.2ZZZ |
| Congestive heart failure | ICD-10 Diagnosis | I25.5 | I25.5ZZZ |
| Congestive heart failure | ICD-10 Diagnosis | I42.0 | I42.0ZZZ |
| Congestive heart failure | ICD-10 Diagnosis | I42.5 | I42.ZZZZ |
| Congestive heart failure | ICD-10 Diagnosis | I43 | I43.ZZZZ |
| Congestive heart failure | ICD-10 Diagnosis | I50 | I50.ZZZZ |
| Congestive heart failure | ICD-10 Diagnosis | P29.0 | P29.0ZZZ |
| Cancer | ICD-9 Diagnosis | 140 | 172.99 |
| Cancer | ICD-9 Diagnosis | 174 | 195.99 |
| Cancer | ICD-10 Diagnosis | C00 | C26.ZZZZ |
| Cancer | ICD-10 Diagnosis | C30 | C34.ZZZZ |
| Cancer | ICD-10 Diagnosis | C37 | C41.ZZZZ |
| Cancer | ICD-10 Diagnosis | C43 | C43.ZZZZ |
| Cancer | ICD-10 Diagnosis | C45 | C58.ZZZZ |
| Cancer | ICD-10 Diagnosis | C60 | C76.ZZZZ |
| Cancer | ICD-10 Diagnosis | C81 | C85.ZZZZ |
| Cancer | ICD-10 Diagnosis | C88 | C88.ZZZZ |
| Cancer | ICD-10 Diagnosis | C90 | C97.ZZZZ |
| Diabetes | ICD-9 Diagnosis | 250.4 | 250.99 |
| Diabetes | ICD-10 Diagnosis | E10.2 | E10.5ZZZ |
| Diabetes | ICD-10 Diagnosis | E10.7 | E10.7ZZZ |
| Diabetes | ICD-10 Diagnosis | E11.2 | E11.5ZZZ |
| Diabetes | ICD-10 Diagnosis | E11.7 | E11.7ZZZ |
| Diabetes | ICD-10 Diagnosis | E12.2 | E12.5ZZZ |
| Diabetes | ICD-10 Diagnosis | E12.7 | E12.7ZZZ |
| Diabetes | ICD-10 Diagnosis | E13.2 | E13.5ZZZ |
| Diabetes | ICD-10 Diagnosis | E13.7 | E13.7ZZZ |
| Diabetes | ICD-10 Diagnosis | E14.2 | E14.5ZZZ |
| Diabetes | ICD-10 Diagnosis | E14.7 | E14.7ZZZ |
| Diabetes | ICD-9 Diagnosis | 250 | 250.3 |
| Diabetes | ICD-10 Diagnosis | E10.0 | E10.0ZZZ |
| Diabetes | ICD-10 Diagnosis | E10.1 | E10.1ZZZ |
| Diabetes | ICD-10 Diagnosis | E10.6 | E10.6ZZZ |
| Diabetes | ICD-10 Diagnosis | E10.8 | E10.8ZZZ |
| Diabetes | ICD-10 Diagnosis | E10.9 | E10.ZZZZ |
| Diabetes | ICD-10 Diagnosis | E11.0 | E11.0ZZZ |
| Diabetes | ICD-10 Diagnosis | E11.1 | E11.1ZZZ |
| Diabetes | ICD-10 Diagnosis | E11.6 | E11.6ZZZ |
| Diabetes | ICD-10 Diagnosis | E11.8 | E11.8ZZZ |
| Diabetes | ICD-10 Diagnosis | E11.9 | E11.ZZZZ |
| Diabetes | ICD-10 Diagnosis | E12.0 | E12.0ZZZ |
| Diabetes | ICD-10 Diagnosis | E12.1 | E12.1ZZZ |
| Diabetes | ICD-10 Diagnosis | E12.6 | E12.6ZZZ |
| Diabetes | ICD-10 Diagnosis | E12.8 | E12.8ZZZ |
| Diabetes | ICD-10 Diagnosis | E12.9 | E12.ZZZZ |
| Diabetes | ICD-10 Diagnosis | E13.0 | E13.0ZZZ |
| Diabetes | ICD-10 Diagnosis | E13.1 | E13.1ZZZ |
| Diabetes | ICD-10 Diagnosis | E13.6 | E13.6ZZZ |
| Diabetes | ICD-10 Diagnosis | E13.8 | E13.8ZZZ |
| Diabetes | ICD-10 Diagnosis | E13.9 | E13.ZZZZ |
| Diabetes | ICD-10 Diagnosis | E14.0 | E14.0ZZZ |
| Diabetes | ICD-10 Diagnosis | E14.1 | E14.1ZZZ |
| Diabetes | ICD-10 Diagnosis | E14.6 | E14.6ZZZ |
| Diabetes | ICD-10 Diagnosis | E14.8 | E14.8ZZZ |
| Diabetes | ICD-10 Diagnosis | E14.9 | E14.ZZZZ |
| Cerebrovascular disease | ICD-9 Diagnosis | 362.4 | 362.4 |
| Cerebrovascular disease | ICD-9 Diagnosis | 430 | 438 |
| Cerebrovascular disease | ICD-9 Diagnosis | 781.4 | 781.4 |
| Cerebrovascular disease | ICD-9 Diagnosis | 782.4 | 782.4 |
| Cerebrovascular disease | ICD-9 Diagnosis | 784.3 | 784.3 |
| Cerebrovascular disease | ICD-9 Diagnosis | 997 | 997 |
| Cerebrovascular disease | ICD-10 Diagnosis | G45 | G45.ZZZZ |
| Cerebrovascular disease | ICD-10 Diagnosis | G46 | G46.ZZZZ |
| Cerebrovascular disease | ICD-10 Diagnosis | H34.0 | H34.0ZZZ |
| Cerebrovascular disease | ICD-10 Diagnosis | I60 | I69.ZZZZ |
| Myocardial infarction | ICD-9 Diagnosis | 410 | 411 |
| Myocardial infarction | ICD-9 Diagnosis | 412 | 412 |
| Myocardial infarction | ICD-9 Diagnosis | 414 | 414.99 |
| Myocardial infarction | ICD-10 Diagnosis | I21 | I22.ZZZZ |
| Myocardial infarction | ICD-10 Diagnosis | I25.2 | I25.2ZZZ |
| Cardiac dysrhythmias | ICD-9 Diagnosis | 427.0 | 427.9 |
| Cardiac dysrhythmias | ICD-9 Diagnosis | 785.0 | 785.1 |
| Cardiac dysrhythmias | ICD-10 Diagnosis | I47.0 | I47.99 |
| Cardiac dysrhythmias | ICD-10 Diagnosis | R00.0 | R00.2 |
| Coronary artery disease | ICD-9 Diagnosis | 411.0 | 411.89 |
| Coronary artery disease | ICD-9 Diagnosis | 413.0 | 413.9 |
| Coronary artery disease | ICD-9 Diagnosis | 414.0 | 414.9 |
| Coronary artery disease | ICD-9 Diagnosis | V458.1 | V458.2 |
| Coronary artery disease | ICD-10 Diagnosis | I20.0 | I20.9 |
| Coronary artery disease | ICD-10 Diagnosis | I23.7 | I23.7 |
| Coronary artery disease | ICD-10 Diagnosis | I24.0 | I24.9 |
| Coronary artery disease | ICD-10 Diagnosis | I25.10 | I25.9 |
| Coronary artery disease | ICD-10 Diagnosis | Z95.1 | Z95.1 |
| Coronary artery disease | ICD-10 Diagnosis | Z95.5 | Z95.5 |
| Coronary artery disease | ICD-10 Diagnosis | Z98.61 | Z98.61 |

CM, Clinical Modification; ICD-9, International Classification of Diseases, Ninth Revision; ICD-10, ICD, Tenth Revision.
