# Supplementary material for: Hyperkalemia treatment modalities: A descriptive observational study focused on medication and healthcare resource utilization
Source: PLoS One. 2020 Jan 7;15(1):e0226844. doi: 10.1371/journal.pone.0226844 (PMC6946143; doi:10.1371/journal.pone.0226844)
Supplement: S3 Table — NoKb, no potassium binder; SPS, sodium polystyrene sulfonate. (DOCX) [file pone.0226844.s005.docx]

# S3 Table. Patients included/excluded from cohort entry.

|  | **Inclusion/exclusion criteria** | **Patiromer** | | **SPS** | | **NoKb** | |
| --- | --- | --- | --- | --- | --- | --- | --- |
|  |  | **n Included** | **% Excluded** | **n Included** | **% Excluded** | **n Included** | **% Excluded** |
| Base cohort | ≥ 1 dispensing or diagnosis code during study period | 1723 | n/a | 20,642 | n/a | 169,337 | n/a |
| AND | Available K^+^ test value 3 months before the index date | 940 | 45 | 8456 | 59 | 53,768 | 68 |
| AND | K^+^ ≥ 5.0 mmol/L 3 months before the index date | 855 | 9 | 7666 | 9 | 35,782 | 33 |
| AND | Medicare Advantage Insurance | 723 | 15 | 6722 | 12 | 26,313 | 26 |
| AND | 6 months of continuous insurance enrollment before index date | 610 | 16 | 5556 | 17 | 21,282 | 19 |
|  | **Total number of patients included and % excluded** | **610** | **65** | **5556** | **73** | **21,282** | **87** |

NoKb, no potassium binder; SPS, sodium polystyrene sulfonate.
