# Supplementary material for: Hyperkalemia treatment modalities: A descriptive observational study focused on medication and healthcare resource utilization
Source: PLoS One. 2020 Jan 7;15(1):e0226844. doi: 10.1371/journal.pone.0226844 (PMC6946143; doi:10.1371/journal.pone.0226844)
Supplement: S4 Table — b/f fu6, before the 6-month follow-up; K+, potassium; NoKb, no K+ binder; SPS, sodium polystyrene sulfonate. (DOCX) [file pone.0226844.s006.docx]

# S4 Table. Attrition table at 6 months post-index.

| **Attrition Table** | **Patiromer** | **SPS** | **NoKb** |
| --- | --- | --- | --- |
|  | **Freq (%)** | **Freq (%)** | **Freq (%)** |
| ***Included in 6-month analyses*** | *106 (17.38)* | *69 (1.24)* | *12,596 (59.19)* |
| ***Censored prior to end of 6-month follow-up period*** | *504 (82.62)* | *5487 (98.76)* | *8686 (40.81)* |
| Loss of insurance b/f fu6 end | 8 (1.31) | 16 (0.29) | 1481 (6.96) |
| Index K^+^ binder discontinued b/f fu6 end | 323 (52.95) | 5361 (96.49) | 0 (0.00) |
| Opposing K^+^ binder start b/f fu6 end | 40 (6.56) | 25 (0.45) | 1361 (6.40) |
| Death b/f fu6 end | 1 (0.16) | 3 (0.05) | 403 (1.89) |
| End of study period b/f fu6 end | 132 (21.64) | 82 (1.48) | 5441 (25.57) |
| **Total** | 610 (100.00) | 5556 (100.00) | 21,282 (100.00) |
| ***Included in 6-month analyses*** | *339 (55.57)* | *3785 (68.12)* | *13,598 (63.89)* |
| ***Censored prior to end of 6-month follow-up period*** | *271 (44.43)* | *1771 (31.88)* | *7684 (36.11)* |
| Loss of insurance b/f fu6 end | 22 (3.61) | 325 (5.85) | 1547 (7.27) |
| Death b/f fu6 end | 0 (0.00) | 43 (0.77) | 333 (1.56) |
| End of study period b/f fu6 end | 249 (40.82) | 1403 (25.25) | 5804 (27.27) |
| **Total** | 610 (100.00) | 5556 (100.00) | 21,282 (100.00) |

b/f fu6, before the 6-month follow-up; K^+^, potassium; NoKb, no K^+^ binder; SPS, sodium polystyrene sulfonate.
